# Autoencoder-based detection of the residues involved in G protein-coupled receptor signaling

**DOI:** 10.1038/s41598-021-99019-z

**Published:** 2021-10-06

**Authors:** Yuko Tsuchiya, Kei Taneishi, Yasushige Yonezawa

**Affiliations:** 1 Artificial Intelligence Research Center, National Institute of Advanced Industrial Science and Technology, 2-4-7 Aomi, Koto-ku, Tokyo, 135-0064 Japan; 2 Center for Advanced Photonics, RIKEN, 2-1 Hirosawa, Wako, Saitama 351-0198 Japan; 3 High Pressure Protein Research Center, Institute of Advanced Technology, Kindai University, 930 Nishimitani, Kinokawa, Wakayama 649-6493 Japan

**Keywords:** Computational biophysics, Machine learning, Computational biology and bioinformatics, Protein function predictions

## Abstract

Regulator binding and mutations alter protein dynamics. The transmission of the signal of these alterations to distant sites through protein motion results in changes in protein expression and cell function. The detection of residues involved in signal transmission contributes to an elucidation of the mechanisms underlying processes as vast as cellular function and disease pathogenesis. We developed an autoencoder (AE) based method that detects residues essential for signaling by comparing the fluctuation data, particularly the time fluctuation of the side-chain distances between residues, during molecular dynamics simulations between the ligand-bound and -unbound forms or wild-type and mutant forms of proteins. Here, the AE-based method was applied to the G protein-coupled receptor (GPCR) system, particularly a class A-type GPCR, CXCR4, to detect the essential residues involved in signaling. Among the residues involved in the signaling of the homolog CXCR2, which were extracted from the literature based on the complex structures of the ligand and G protein, our method could detect more than half of the essential residues involved in G protein signaling, including those spanning the fifth and sixth transmembrane helices in the intracellular region, despite the lack of information regarding the interaction with G protein in our CXCR4 models.

## Introduction

Ligand binding induces changes in the protein dynamics by altering the fluctuations in mainly side-chain atoms of the residues involved in ligand binding. The signal of this change, particularly the changes in side-chain fluctuations, is transmitted from the ligand-binding site to distant regions (namely, allosteric sites) through protein motions, and to downstream proteins through various interactions, which result in changes in protein expression and cell function^1–7^. A better understanding of this phenomenon, i.e., signal transmission, contributes to an elucidation of the mechanisms of cellular functions and the onset of disease. In our previous study, we developed a method for detecting the residues involved in signal transmission by analyzing the difference in patterns of fluctuations in a residue pair between ligand-bound (holo) and unbound (apo) forms^8^. With this method, the fluctuation data obtained through molecular dynamics (MD) simulations of the protein in apo and holo forms were compared in terms of the similarity of the fluctuation patterns using an unsupervised neural network, an autoencoder (AE), and the residues essential in signaling were detected based on the results of the inspection using the AE. In the previous study, we focused on the detection of essential residues involved in the transmission of the signal of ligand binding to allosteric sites without large conformational deformations in PDZ2 protein^8^. This event, which is mainly caused by changes in the side-chain conformations, is known as dynamic allostery^1–7^. The dynamic allostery in PDZ2 has been analyzed through several computational studies based on MD simulations of ligand-bound and -unbound PDZ2 proteins, where the analyses of the degree of conformational fluctuations and the correlation between the fluctuations of two residues revealed key residues for dynamic allostery in PDZ2^9–12^. However, we used the AE to analyze the dynamic allostery by inputting the vector of the side-chain distances between the residues during the simulation, namely, the data of the time fluctuations of the side-chain distances in a pair of residues. This method can detect most of the essential residues involved in the signaling in PDZ2 that were detected through an NMR study^13^, and the number of detected residues was larger than that in other MD studies^9–12^, which focused on the changes in the degree of conformational fluctuation and correlation between fluctuations of two residues through ligand binding. Their ideas are completely different from ours, which focuses on the changes in protein fluctuations without large conformational changes owing to ligand binding.

As another important difference from previous MD studies, we did not perform the averaging of the structural coordinates. MD simulations were used to calculate the trajectories of the residues and atoms within a specified time frame. The mechanisms of dynamics changes and signal transduction can be analyzed by extracting the degree of conformational fluctuations and the correlation of the fluctuations between residues from the trajectories. In general, these analyses require averaging the structural coordinates in the trajectories through least-squares fitting. However, this may disregard subtle changes in the conformations, particularly side-chain conformations, between the apo and holo forms. To avoid this concern and acquire all of the important information, we used the AE in which the time fluctuations of the side-chain conformations in a pair of residues during the simulations were compared between the apo and holo forms using the distance data as the input vectors for the AE. As a result, averaging is unnecessary.

In this paper, we introduce the application of the AE-based method to the analysis of G protein-coupled receptor (GPCR) signaling to confirm whether the method can detect essential residues involved in such a large system. In GPCRs, which are a large and diverse protein family encoded in the human genome^14^, ligand binding that occurs in the extracellular (EC) region leads to conformational changes in the fifth, sixth, and seventh transmembrane (TM) helices^15^. This signal is transmitted to the intracellular (IC) region through the TM helices, which leads to interactions with G proteins in a cell. In this study, we attempted to detect the essential residues involved in the transmission of ligand-binding signals to G proteins through TM regions. Herein, we focused on C-X-C chemokine receptor type 4 (CXCR4), a member of the class A-type GPCRs. Chemokine receptors are involved in the stimulation of cell migration^16^; in particular, CXCR4 is involved in tumor progression, angiogenesis, metastasis, and survival^17^. Therefore, an analysis of CXCR4 signaling is essential to elucidate the mechanism of tumor metastasis. We first conducted MD simulations of the CXCR4 structure with and without a ligand, using the crystal structure of CXCR4 in complex with the chemokine vMIP-II (Protein Data Bank [PDB] ID: 4rws)^18^ used as the initial model. In addition, using our AE-based method, we analyzed the signaling by detecting the residues essential for the transmission of the signal from the ligand to the G protein from the MD trajectories.

To confirm the accuracy of the AE-based detection, we referred to the structure of C-X-C chemokine receptor 2 (CXCR2) in complex with the chemokine ligand; interleukin-8 (IL8), known as CXCL8; and the G protein determined through cryo-electron microscopy (EM) (PDB IDs: 6lfm and 6lfo)^19^. We extracted 25 residues described in the literature as being essential for ligand binding, conformational changes of the TM helices induced through ligand binding, and the interaction and activation of the G protein. Our AE-based method was able to detect more than half of these essential residues involved in signaling in CXCR2; the method detected the essential residues involved in the change of fluctuations by ligand binding, containing the residues in the IC regions involved in signaling to the G protein, despite the lack of information on the interaction between CXCR4 and the G protein in our models, whereas the method did not detect the residues involved in conformational changes by ligand binding. It implies that our AE-based method evaluated the change in fluctuations, and not the conformations, induced through ligand binding. We also applied widely used methods for analyzing the correlative fluctuations from the MD trajectories, conducting a principal component analysis (PCA) and calculating the dynamic cross-correlation matrices (DCCMs), and then compared the correlative fluctuations between the apo and holo forms. The results of the PCA and DCCM analyses of our data showed the high sensitivity of these methods to conformational changes induced through ligand binding in GPCR protein, rather than to the signaling. These observations imply that our AE-based method can be utilized to elucidate signaling mechanisms in many different systems, particularly those of disease onset.

## Results and discussion

**Figure 1:**
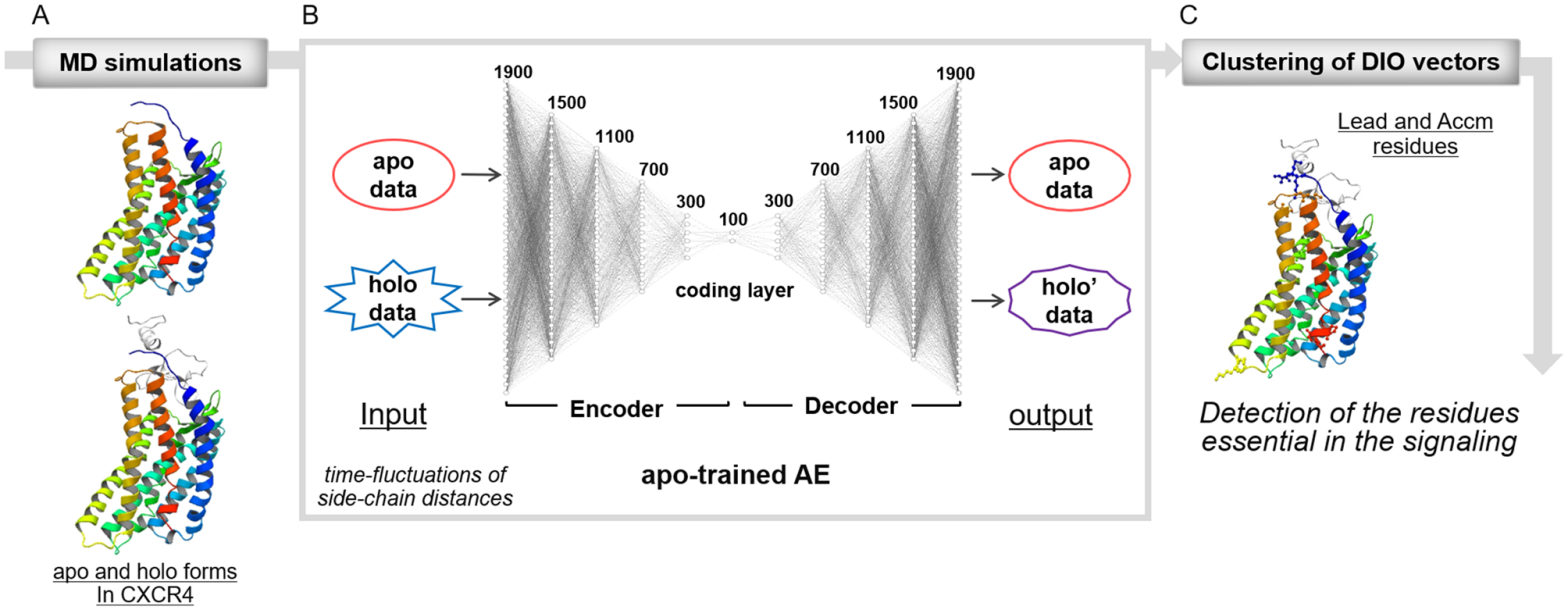
Architecture of autoencoder-based detection of residues essential in signaling. **(A)** Executions of the MD simulations of apo and holo forms. **(B)** Training of AE using apo data, and the inspections of apo and holo data using the apo-trained AE. The AE consists of a six-layered encoder and decoder, and a coding layer. The vectors of the side-chain distances during the simulations were inputted into the AE. The output from the inspection of the apo data was almost the same as the input data, whereas that of the holo data showed differences from the input data because of the modification according to the apo features. **(C)** The clustering of the differences between input and output (DIO) vectors used to extract the residues essential in signaling, as Lead and Accm residues.

The architecture of our AE-based method is shown in Fig. 1.

### Construction of CXCR4 apo structures through MD simulation

1. **Comparison of RMSFs in the apo and holo forms**. In this study, we focused on the signal transmission initiated by ligand binding in GPCR CXCR4. We used the crystal structure of CXCR4 bound to the viral chemokine vMIP-II (holo form, PDB ID: 4rws)^18^. Because a CXCR4 structure without a ligand (apo form) has yet to be determined experimentally, we constructed a model structure in the apo form from the CXCR4 structure in the holo form by executing MD simulations after eliminating the ligand structure, as described in the Methods section. To confirm whether the structural models in the apo form were “apo-like” and differ from those in the holo form, we calculated the RMSF for the trajectories, which is a standard measure of fluctuations in each residue during the simulation (Supplementary Fig. S1A online). The turn in the IC region between the fifth and sixth TM helices (hereafter, the fifth and sixth TM helices are referred to as TM5 and TM6, respectively, as shown in Fig. 2, where the definitions of the helix and turn regions (colored from blue to red) were described) had large RMSF values of Cα atoms in apo1, holo1, and holo2. This turn was involved in the conformational changes in TM5 and TM6 through ligand binding, and is also known to be involved in the signal transmission to the G protein. They suggest that the conformations around the turn between TM5 and TM6 were more flexible in holo form than in apo form. The RMSDs of the Cα atoms around the turn between TM5 and TM6 indicated that the structural transition at approximately 700 ns, rather than large fluctuations during the simulation, arose only in the apo1 structure (Supplementary Fig. S1B online). This might lead to a large RMSF around the TM5–TM6 turn. By contrast, the TM5–TM6 turns in holo1 and holo2 had large RMSFs and stable RMSDs. These observations suggest that the fluctuation patterns in the TM5–TM6 turn regions in holo form were unique and deferred from those in the apo form, which might regulate the transmission of the signal to the G protein. The RMSFs in the turn between TM4 and TM5, which are involved in ligand binding, were slightly larger in the apo (apo1 and apo2) forms than in the holo (holo1 and holo2) forms, indicating conformational freedom through the removal of the ligand.

**Figure 2:**
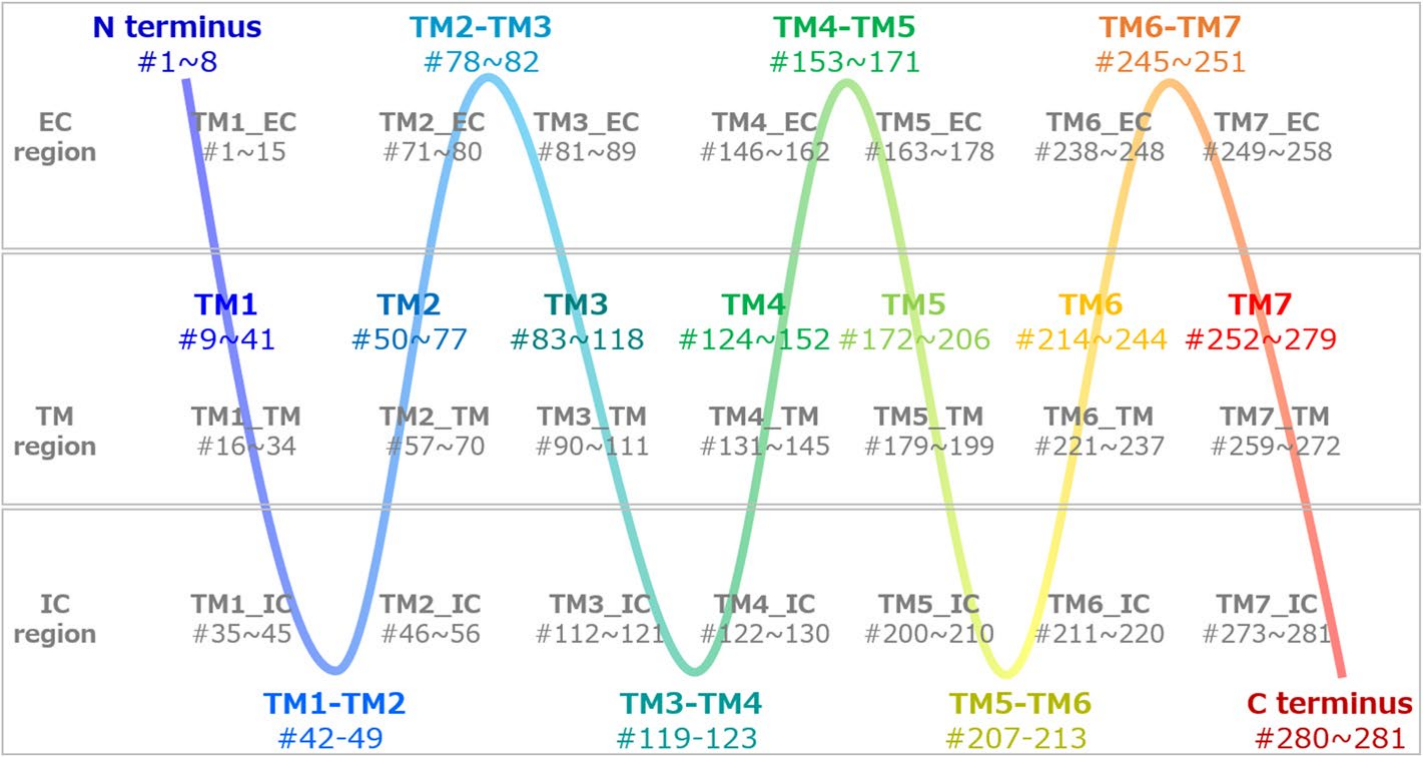
Definition of EC, IC, and TM regions. Based on the crystal structures of CXCR4 (PDB ID: 4rws)^18^ and the snapshots of the MD simulations, we defined the structural regions of the TM helices as residue numbers from 9 to 41 (TM1), from 50 to 77 (TM2), from 83 to 118 (TM3), from 124 to 152 (TM4), from 172 to 206 (TM5), from 214 to 244 (TM6), and from 252 to 279 (TM7 helix regions). The other residues are located in the N-terminus (from 1 to 8), C-terminus regions (from 280 to 281), or turn regions (e.g., the turn between TM1 and TM2 helices [TM1–TM2]). This definition is gradually represented from blue to red. To examine which function (and region), i.e., the signaling from the ligand (EC region), the signaling to the G protein (IC region), or the transmission of the signal from EC to IC regions (TM region), the important residues were involved in, we segmented 1 helix (e.g., TM1, the residue numbers from 9 to 41) into 3 regions, EC, IC, and TM. First, we defined the TM region, which consisted of residues located from the 8th residue from the N-terminus to the 8th residue before the C-terminus of TM1 (TM1_TM, the residue numbers from 16 to 34). The EC region consisted of residues located before the 8th residue from the N-terminus of TM1 (the residue numbers from 9 to 15) and N-terminus (from 1 to 8). The IC region consisted of the residues located after the 8th residue from the C-terminus of TM1 (from 35 to 41) and the former residues of the TM1–TM2 turn from the middle (from 42 to 45). Thus, TM1_EC and TM1_IC consisted of the residues with the numbers from 1 to 15, and from 35 to 45, respectively. TM2_IC, therefore, consisted of the residues with the numbers from 46 to 56, where the numbers from 46 to 49 were from the TM1–TM2 turn, and those from 50 to 56 were from TM2. TM7_IC also included the residues with the numbers after the 8th residue from the C-terminus of TM7 (from 273 to 279) and the C-terminus (from 280 to 281). This definition is indicated in gray.
2. **Comparison of intra-receptor protein contacts in the apo and holo forms**. We also compared the intra-receptor protein residue contacts in the structural models of the apo and holo forms. We defined a residue pair with the distance between any atoms in two receptor residues shorter than 4 Å as the pair forming an intra-receptor contact. The total number of intra-receptor contacts during the simulations in apo form (19,120 and 19,104 contacts in the apo1 and apo2 data, respectively) was larger than that in holo form (16,823 and 16,761 contacts in the holo1 and holo2 data, respectively).
  To analyze these contacts more accurately, we classified them based on the helix numbers (from TM1 to TM7) and the regions on the helix (extracellular (EC), transmembrane (TM), and intercellular (IC) regions), such as “TM1_EC”, as written in gray in Fig. 2. This classification is defined in the legend of Fig. 2. The numbers of intra-receptor contacts were added together in each helix region (e.g. “TM1_EC”), as summarized in Supplementary Table S1 online. We focused on the following four pairs of helix regions, TM6_TM and TM7_TM, TM5_EC and TM6_EC, TM3_IC and TM6_IC, and TM2_TM and TM7_IC, where the number of contacts between the two helix regions was greater than 100 in both apo1 and apo2 forms, or both holo1 and holo2 forms, and the ratios of the numbers of contacts between apo and holo forms, more precisely, apo1 and holo1, apo2 and holo1, apo1 and holo2, and apo2 and holo2 forms, were smaller than 0.1, where the indicates the quotient of the smaller number ratio divided by the larger numbers (Supplementary Table S1 online).
  In the pairs between TM6_TM and TM7_TM, and between TM5_EC and TM6_EC, the number of contacts in apo forms was larger than that in holo forms. The EC regions of the helices were narrowed by the conformational changes of helices in apo form, as shown in Supplementary Fig. S2A online. We suppose that the elimination of the ligand led TM5, TM6, and TM7 to return the structural conformations. As a result, TM5_EC and TM6_EC came close to each other, leading to the bending of TM6 and TM7, which also led to the formation of contacts between TM6_TM and TM7_TM. By contrast, the pairs between TM3_IC and TM6_IC, and those between TM2_TM and TM7_IC, have larger numbers of contacts in holo forms than in apo forms. As shown in Supplementary Fig. S2B online, TM3_IC and TM6_IC, and TM2_TM and TM7_IC, were close to each other in holo form, whereas they separated from each other in apo form. These observations also indicate that the elimination of the ligand led to conformational changes in the TM6 and TM7 helices in the IC region.
  We speculate that the apo structure of GPCR is flexible and does not have a stable conformation. Therefore, we believe that the difference between intra-receptor protein contacts in apo and holo forms and the conformational changes by eliminating the ligand might be sufficient to explain the formation of apo structures. Based on the comparisons of the RMSFs and intra-receptor protein contacts between the apo and holo forms, we believe that the MD simulations of the crystal structure without the ligand could eliminate the effect of ligand binding and allow the construction of an apo(-like) structure of CXCR4.

### AE-based comparison of MD trajectories between the apo and holo forms

To find clues for understanding the mechanism of signaling in CXCR4, we compared the MD trajectories to identify differences in the fluctuation patterns between the apo and holo forms using the characteristics of AE: reconstruction of the input data by extracting the features in the input through a dimensionality reduction^20^. The vector of the side-chain distances in a pair of residues during the simulation was used as input data for the AE because we believed that the detection of subtle changes in the side-chain conformations and fluctuations was required in the analyses of the signal transmission from the ligand to the G protein. The distance vectors were constructed directly from the trajectory data. Thus, an averaging of the structural coordinates is not executed. A set of vectors in all residue pairs was used as the AE input data. We trained two AEs, apo1- and apo2-trained AEs, using a set of distance vectors in the apo1 and apo2 data, respectively. The apo1-trained AE was used to inspect the apo1, holo1, and holo2 data. Similarly, the apo2-trained AE was used to inspect the apo2, holo1, and holo2 data.

The output from the inspection of the apo data was almost the same as the input data because of the correct reconstruction using the AE (Fig. 1). However, the output from the inspection of the holo data showed “differences” from the input data in partial or whole time steps because the output data were more or less modified from the input data according to the features of the apo form. This indicates that the residue pairs with “differences” were involved in different patterns of fluctuations from those in the apo data in the corresponding time steps. To extract the different patterns of fluctuations, we took the difference between the input and output (DIO) vectors, as described in the Methods section.

### Clustering of DIO vectors

To extract the residues in holo form that were involved in the patterns of fluctuations different from those in apo form, that is, those involved in the transmission of the signal through ligand binding, we conducted a clustering of the residue pairs based on the vectors of DIO data, as described in the Methods section. The four combinations of apo and holo data were clustered based on the DIO vectors: combinations of apo1 and holo1 data, and apo1 and holo2 data, which were obtained through the inspections using apo1-trained AE, and those of apo2 and holo1 data, and apo2 and holo2 data, obtained through the inspections using apo2-trained AE. In this study, the clustering of the DIO vectors divided the receptor–receptor, receptor–ligand, and ligand-ligand residue pairs in the apo and holo forms simultaneously into nine groups. Among all combinations of the apo and holo forms, all residue pairs in apo form were clustered into one or two large groups during the early clustering steps. The residue pairs in the holo form were separated (Supplementary Fig. S3A-S3D online). We defined a residue that formed pairs with more than or equal to 80% of other residues as a “leading (Lead) residue,” which may lead to cluster-specific fluctuations and their transmissions. In addition, a residue that forms pairs with more than or equal to 60% and less than 80% of other residues was defined as an “accompanying (Accm) residue,” which may assist in leading and transmitting cluster-specific fluctuations^8^. Notably, we stopped at the ninth clustering because we cloud not obtain additional Lead and Accm receptor residues in the tenth clustering, and we did not focus on the Lead and Accm residues in the ninth cluster because this cluster included many remaining residue pairs. In addition, although we clustered all receptor–receptor, receptor–ligand, and ligand–ligand residue pairs, only the Lead and Accm receptor residues were the focus of this study.

A total of 102 Lead and/or Accm residues were identified, 73 of which were found in all or several of the four combinations of apo and holo data, and the other 29 residues were identified as Lead or Accm residues depending on the combination (Supplementary Table S2 online, where the numbers of Lead and Accm residues and the total numbers without overlaps in each combination are shown). The numbers of Lead and/or Accm residues identified from receptor–receptor and receptor–ligand residue pairs were 33 and 30, respectively, and the other 39 were from both receptor–receptor and receptor–ligand residue pairs. Thus, the Lead and Accm residues overlap between two or more combinations and in receptor–receptor and receptor–ligand residue pairs. Moreover, the total numbers of these residues varied in the four combinations, from 37 in the apo2–holo1 to 76 in the apo2–holo2 combinations.

**Figure 3:**
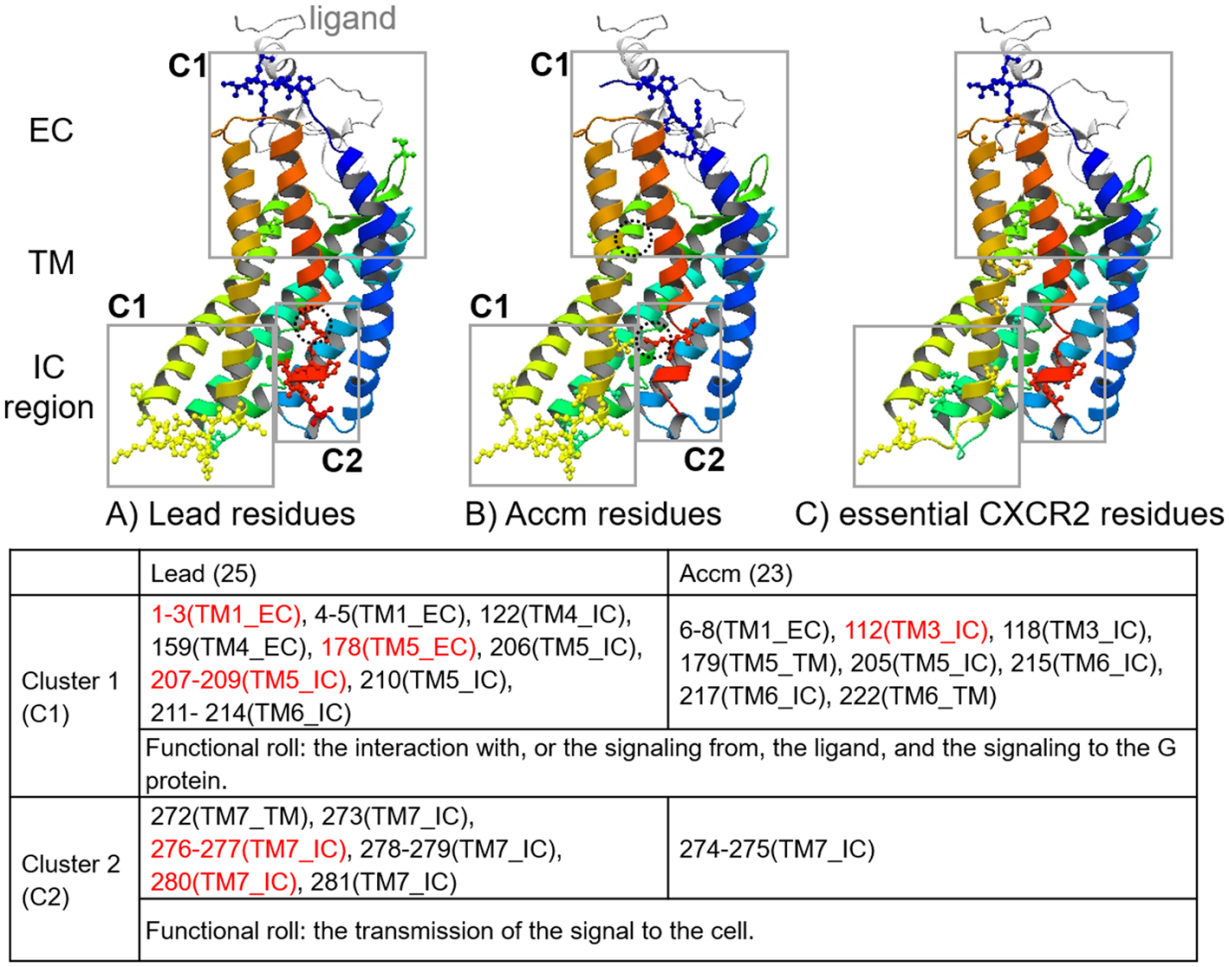
Lead and Accm residues in CXCR4 corresponding with the essential residues in CXCR2 signaling. **(A)** The Lead residues in CXCR4 in the apo2–holo1 combination. **(B)** The Accm residues in CXCR4 in the apo2–holo1 combination. **(C)** The essential CXCR2 residues in the signaling. (**A–C)** All residues are indicated using ball and stick models on the snapshot at 1000 ns in the CXCR4 holo1 structure, where CXCR4 is gradually colored from blue (N-terminus) to red (C-terminus) and the ligand residue is colored gray. **(A,B)** The Lead **(A)** and Accm **(B)** residues in the gray boxes with “C1” and “C2” belong to Clusters 1 and 2, respectively. The residues in the TM regions are circled by dotted lines in black. **(C)** The 25 essential CXCR2 residues in the signaling were extracted from the literature based on the cryo-EM structures of CXCR2-ligand-G protein complexes^19^ (Table). The Lead and Accm residues and the functional rolls in apo2–holo1 combination. The residue numbers, the helix numbers, and the regions on the helix of the Lead and Accm residues in Clusters 1 and 2 and their functional roles are summarized. The Lead and Accm residues that were consistent with the essential CXCR2 residues are colored red.

To examine which function (and region), the signaling from the ligand (EC region), the signaling to the G protein (IC region), or the transmission of the signal from the EC to IC regions (TM region), the Lead and Accm residues were involved in, we classified the Lead and Accm residues into three regions (e.g., TM1_EC, TM1_IC, or TM1_TM regions), as described in Fig. 2. In the four combinations, clusters exist, including Lead and Accm residues in only the EC or IC region, and residues in the EC and TM, IC and TM, and all three regions. Interestingly, the apo2–holo1 combination had a cluster that included the residues in all three regions and had the smallest number of both Lead and Accm residues in all four combinations (Fig. 3).

In all four combinations, 47, 19, and 36 residues in the EC, TM, and IC regions, respectively, were assigned as Lead and/or Accm residues (Supplementary Table S2 online). Most of the 19 TM residues were identified as Accm (17 residues, 2 of which were also identified as Lead residues). In addition, 16 of them came from receptor–ligand residue pairs, 3 of which were also from receptor–receptor residue pairs. By contrast, all of the 36 Lead and/or Accm residues in the IC regions came from receptor–receptor residue pairs, of which 2 residues were also from the receptor–ligand pairs. In the EC regions, 21, 17, and 9 Lead and/or Accm residues from receptor–receptor, receptor–ligand, and both receptor–receptor and receptor–ligand residue pairs were observed. These observations suggest that the Accm residues in the TM region assisted in a signal transmission from the ligand to the IC region, and the Lead residues in the IC residues were strongly involved in the signaling to the G protein.

In this study, the EC residues in the N-terminus and TM1 (TM1_EC) and the IC residues in TM5 and TM6 (TM5_IC and TM6_IC) were detected as Lead and/or Accm residues for all four combinations (Supplementary Table S2 online). The EC residues in TM6 and TM7 (TM6_EC and TM7_EC) in all combinations except apo2–holo1, and the IC residues in TM7 and the C-terminus (TM7_IC) in all combinations except apo1–holo2, were also detected. It is known that the EC regions in TM1, TM4, and TM5 are involved in ligand binding and signaling from the ligand, and that the conformations of the TM5, TM6, and TM7 helices are altered by ligand binding. In addition, the IC regions in TM5 and TM6 (and TM7) are involved in signaling to the G protein (and arrestins). These results imply that our AE-based methods can detect essential residues involved in the transmission of the signal from the ligand to the cell through an interaction with the G protein, particularly within the IC regions of TM5 and TM6.

### Validation of AE-based results through a comparison with the experiment results

The structures of CXCR2 in complex with CXCL8 and the G protein were recently determined using cryo-EM (PDB IDs, 6lfo (monomer ligand) and 6lfm (dimer ligand), respectively)^19^, of which the literature revealed 35 essential residues involved in signaling. We examined whether our AE-based method could detect the essential residues involved in CXCR2 signaling, as Lead and Accm residues in CXCR4. Of note, we eliminated 10 of the 35 essential residues described in the above literature because one residue corresponded with a gap in CXCR4 based on the multiple sequence alignment of CXCR family members (Supplementary Fig. S4 online)^21^, and the remaining 8 and 1 residue were located before the N-terminus and after the C-terminus in our structure model, respectively. Consequently, we compared our results with 25 essential CXCR2 residues, of which 8, 7, 3, and 7 residues were involved in the interaction with, or the signaling from, the ligand (the “Ligand” interaction shown in Table S3), the conformational changes of TM5 and TM6 or the synergetic rearrangement of TM3 (“TM5conf”), the conformational changes of TM7 for arrestin recognition, which might lead the transmission of the signal to the cell (“TM7conf”), and the signaling to the G protein (“G protein”), respectively. Our method detected 14 (56%, of which the recall and precision were 0.56 and 0.14, respectively) of the 25 essential residues as Lead and/or Accm residues. Most of the essential CXCR2 residues involved in “Ligand” and “TM7conf” interactions (seven and three residues, respectively) and approximately half of those involved in the “G protein” interaction (three residues) were consistent with the Lead and/or Accm residues, whereas only one of seven essential CXCR2 residues involved in the “TM5conf” interaction was detected, as summarized in Supplementary Table S3 online, and in Fig. 3, where the result of the apo2-holo1 combination is shown as an example. Thus, our method could detect the residues involved in the signaling from ligand within the EC region (“Ligand” interaction) to the G protein or arrestin within the IC region (“TM7conf” and “G protein” interactions), whereas the method did not detect most of residues involved in the conformational changes by ligand binding (“TM5conf” interaction). Similarly, the other four residues in the “G protein” interactions that were involved in the formation of the hydrophobic pocket to interact with G protein, were also not detected. These findings suggested that our method evaluated the changes in fluctuations induced through ligand binding, rather than conformations. Our CXCR4 model did not include any structural information on the G protein. Therefore, we speculated that the peculiar pattern of fluctuations in the residues in the IC regions of TM5, TM6, and TM7 led to the interaction with the G protein.

From a different perspective, most of the 14 CXCR4 residues that were consistent with the essential CXCR2 residues were detected as Lead residues (six residues detected as only Lead and six detected as both Lead and Accm residues, respectively), and the other two residues were detected as only Accm residues, one of which is the only residue involved in the “TM5conf” interaction. Thus, our AE-based method could detect more than half of the essential residues identified from the experimental CXCR2 structure as Lead and Accm residues in CXCR4, most of which were involved in the signaling from the ligand to the G protein and identified as Lead residues. Both CXCR4 and CXCR2 are members of the C-X-C-chemokine receptor family, and their sequence identity is approximately 37%. Among the 11 essential residues that were extracted from the CXCR2 structures but inconsistent with any Lead or Accm residues in CXCR4, 3 (1 in the EC region of TM5 and 2 in the IC region of TM6) were not conserved in the CXCR family (Supplementary Fig. S4 and Table S3 online). The different types of amino acids may alter these interactions, leading to different fluctuation patterns. If the non-conserved residues in CXCR4 fluctuate in different patterns from the corresponding residues in CXCR2, it may be difficult to detect the non-conserved residues as important residues using our AE-based method.

We also conducted PCA-like analyses and calculations of the DCCMs, which are widely used in the analyses of correlative fluctuations from the trajectories obtained through the MD simulations. PCA elucidates the collective motions of the entire structure, whereas we analyzed the fluctuation patterns of each residue pair during the simulation. Because it is difficult to compare the PCA results directly with our results, we examined the correlation between the average residue–residue distance and the variance of the distance within a range of time steps. As shown in Supplementary Fig. S5 online, the results of the PCA-like analyses may correlate with those of the RMSFs. The DCCM calculates the correlation between fluctuations in the residues in a protein during the simulation. As shown in Supplementary Fig. S6 online, DCCMs based on the motions of Cα atoms in receptor proteins show a sensitivity to conformational differences, particularly through ligand exclusion, rather than through the signaling. These results imply the difficulty of extracting residues or residue pairs involved in signaling from such analyses. In addition, we conducted a network analysis on DCCM and calculated the precision and recall for the experimental results by ranking through the betweenness centrality. As a result, the recall and precision were 0.32 and 0.10, respectively, which confirms the superiority of our AE-based method in this comparison.

In conclusion, the AE-based method could detect more than half of the essential residues involved in signal transmission by a class A-type GPCR, CXCR4, based on the analyses of the changes in the residue fluctuations through ligand binding. This study showed that the Lead residues in the EC regions played a role in the reception of a signal of ligand binding and the transmission of the signal to the IC regions, whereas the Accm residues in the TM region assisted in a signal transmission from the EC to IC regions. The Lead residues in the IC regions transmitted the signal to the G protein in a cell through interactions with the G protein, which was led by fluctuations in the TM5–TM6 turn. Thereafter, the G protein may stimulate downstream proteins in the cell. In this pathway, the signal from the ligand was transmitted into the cell through cooperative fluctuations of the receptor residues, led by Lead and Accm residues extracted from both receptor–receptor and receptor–ligand residue pairs. We believe that our AE-based method can be applied to the detection of essential residues involved in the signal transmission in the mechanisms of diseases, which will help in the development of treatments for diseases and drug discovery.

## Methods

### MD simulations

Structural data for the chemokine receptor CXCR4 in complex with the viral chemokine vMIP-II encoded using Kaposi’s sarcoma-associated herpesvirus (PDB ID, 4rws)^18^ were downloaded from Protein Data Bank Japan^22^. Missing hydrogen atoms were complemented using the pdb2gmx program in the GROMACS program^23^ under pH 7 conditions. The protein structure was inserted into the center of the lipid bilayer, which is composed of phosphatidylcholines (POPCs), discarding overlapping lipid molecules. The protein–membrane system was immersed in a rectangular box of water solvent. Subsequently, sodium and chloride ions were replaced with an adequate amount of water to an ion concentration of 150 mM, with the total charge being neutral. A CHARMM force field (version 27)^24^ was applied to the proteins and lipids. The TIP3P^25^ model was used for water. The system was treated under periodic boundary conditions. The x-, y-, and z-dimensions of the system were 8.6, 8.6, and 10.5 nm, respectively. The electrostatic energy and force were calculated using the particle mesh Ewald method^26^, wherein the cut-off lengths of the short electrostatic calculation and van der Waals interactions were set to 8 Å^27^. The chemical bonds associated with hydrogen atoms were constrained using the LINCS algorithm^28^, and water molecules were restrained through the SETTLE algorithm^29^. The total energy of the system was minimized using the steepest descent method. The energy-minimized system was equilibrated using an MD simulation with the Berendsen algorithm^28^ at 310 K. The thermally equilibrated system was also equilibrated under constant temperature and constant pressure conditions using the Parrinello–Rahman method^30^ at 310 K with a pressure of 1 atm. The time-step length of the MD simulations was set to 2 fs. To investigate the ligand allosteric effects on the chemokine receptors, we prepared holo (a receptor with a chemokine ligand) and apo (a receptor without a chemokine ligand) systems in the same manner. The 1-μs production runs of the systems were conducted twice for the holo and apo forms for confirmation with different initial velocities, and snapshots were collected every 2 ps for the analysis, where we designated the obtained data as apo1, apo2, holo1, and holo2 data. All MD simulations were conducted using GROMACS 4.5.5^23^. The root mean square deviation (RMSD) and root mean square fluctuation (RMSF) were calculated for each of the four trajectories (Supplementary Fig. S1A online).

### Multilayer pyramidal autoencoder

The AE, an unsupervised neural network, is widely used for a dimensional reduction, feature extraction, and pattern recognition^20,31–33^. In this study, we used a multilayer AE comprising 13 fully connected layers (6 layers in the encoder and decoder parts consisting of 400 to 1900 nodes with 300-node increments, and a coding layer comprising 100 nodes) (Fig. 1). The AE constructs a neural network that reconstructs the input and learns through backpropagation to minimize the mean squared error (MSE) between the original input and the reconstructed output. If the reconstruction error is sufficiently minimized, the AE extracts the features of the dynamics of the residue pairs according to the dimensions of the coding layer, which is the central layer, and the output is represented as a composite of the features.

The vector of the side-chain distances between residues was used as the input data. In more detail, the distance between the centers of mass of the side-chain atoms, except for hydrogen atoms, in a pair of residues (i.e., side-chain distance), was calculated from 50.5 to 1000 ns in 0.5-ns increments (1900 steps) within the trajectory obtained through the MD simulation. The distance data were reconstituted as a 1900-dimensional vector for each residue pair. For each of the trajectories in apo1, apo2, holo1, and holo2 data, a set of distance vectors for all residue pairs was prepared (39,340 and 64,261 vectors [residue pairs]) in apo1 and apo2 and holo1 and holo2 data, respectively, wherein the holo1 and holo2 data contained the distance data of the pairs between the receptor and ligand residues and between the ligand and ligand residues.

The AE was trained using 80% of the randomly selected distance vectors (31,472 vectors) in training and 20% (7,868 vectors) for validation of apo1 or apo2 data. Thus, we constructed two AEs: apo1-trained and apo2-trained AEs. In this study, the Adam optimizer with a learning rate of 0.00001 was used. The training data were divided into a set of vectors for every 100 residue pairs (i.e., batch size = 100). The training was iterated for 100,000 epochs and terminated when the validation loss decreased. We decided to use the above-described hyperparameters because of the best performance in terms of loss and accuracy in the training and validation steps among their various combinations. Inspections were then conducted using the apo1-trained AE for apo1, holo1, and holo2 data and apo2-trained AE for apo2, holo1, and holo2 data (Fig. 1).

### Difference between input and output vectors (DIO)

The input vector shows the time fluctuation of protein motions, and the output vector shows the time fluctuation of protein motions exaggerated and modified according to the features of the apo form. DIO revealed a unique pattern of protein motion only in holo form^8^. The DIO vector, i.e., the vector of the difference between elements in the input and output vectors in each time step, was calculated for each residue pair. The frequency of the residue pairs with nonzero DIOs was higher in holo form than in apo form, with most of the residue pairs in the apo form having near-zero DIOs (Supplementary Fig. S3E and S3F online). Therefore, we decided to conduct the analyses of DIOs to detect the unique features of the protein motions observed only in holo form.

### Clustering of residue pairs in receptor proteins

The residue pairs in apo and holo forms were then classified based on the similarity of the motion patterns during the simulation through the clustering of the DIO vectors. We used the “cosine” method of the “dist” function and the “ward.D2” linkage of the “hclust” hierarchical clustering function in the R program^34^ to segment residue pairs based on the cosine similarities among the DIO vectors. To observe the process of segmentation of residue pairs in both apo and holo forms, we used the “cutree” function, which cuts a dendrogram tree into several groups by specifying the desired number of clusters. We executed the clustering of four combinations of the apo and holo forms (apo1 and holo1, apo1 and holo2, apo2 and holo1, and apo2 and holo2) eight times with a cut-off value of 2 to 9 (Supplementary Fig. S3A–S3D online).

We clustered the input and output vectors for confirmation (Supplementary Fig. S3G and S3H online). This revealed no clear separation between the residue pairs in the apo and holo forms, probably because of the much larger absolute values of the elements in the input and output vectors than those in the DIO vectors.

## Supplementary Information

Supplementary Information.

## Data Availability

(1) MD simulations of CXCR4 and CXCR4-ligand complex : (i) Structure data obtained from Protein Data Bank (PDB ID: 4rws), (ii) MD simulations conducted using GROMACS 4.5.5, (iii) RMSD, RMSF, and DCCM calculations using GROMACS 2018. (2) AE-based comparison of the trajectory data using the neural network framework, Chainer 5.3.0. The AE code is available at https://github.com/taneishi/protein_ae. (3) Clustering of DIO vectors using R 3.6.3, (4) Comparison of the predicted results with the EM structure of CXCR2 (PDB IDs: 6lfo and 6lfm), (5) Sequence alignments of CXCR family using the GPCRdb web server, (6) PCA analyses using MODE-TASK program.

## Abbreviations

AE: Autoencoder
MD: Molecular dynamics
GPCR: G protein-coupled receptor
EC: Extracellular
TM: Transmembrane
IC: Intracellular
PDB: Protein data bank
EM: Electron microscopy
RMSD: Root mean square deviation
RMSF: Root mean square fluctuation
MSE: Mean squared error
