# Supplementary material for: Autoencoder-based detection of the residues involved in G protein-coupled receptor signaling
Source: Sci Rep. 2021 Oct 6;11:19867. doi: 10.1038/s41598-021-99019-z (PMC8494915; doi:10.1038/s41598-021-99019-z)
Supplement: Supplementary file 1 — Supplementary Information. [file 41598_2021_99019_MOESM1_ESM.pdf]

Autoencoder-based detection of the residues involved in G protein-coupled receptor signaling

Yuko Tsuchiya, Kei Taneishi, Yasushige Yonezawa

Supplementary Information

Figure S1 Root mean square deviations and fluctuations in CXCR4

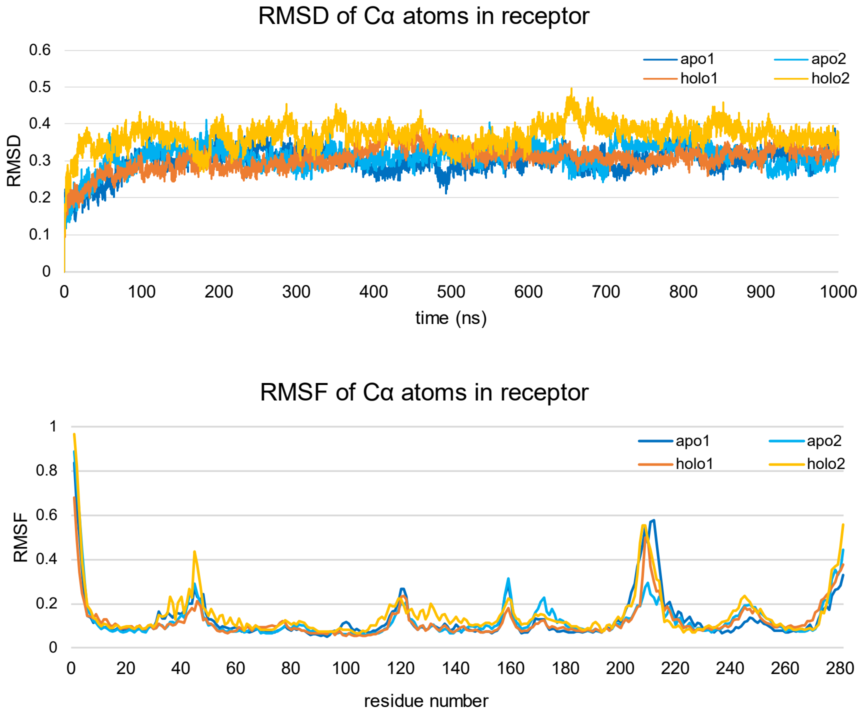

A)

A) Root mean square deviation (RMSD) and fluctuation (RMSF)

The root mean square deviation (RMSD) was used as a standard measure of similarity between structures. The RMSD is defined as follows:

$$RMSD = \sqrt{\frac{1}{N}\sum_i (r_i - r_i^{ref})^2}$$

where i is the i-th C_α atom, N is the number of C_α atoms, r_i is a state vector of the MD trajectory, and r^{ref} is the position vector of a reference structure. r_i was fitted to r_i^{ref} by a least-squares regression. First structure of the analyzed trajectory was set to the reference structure in this study.

The root mean square fluctuation (RMSF) of the i-th C_α atoms was calculated using the following equation:

$$RMSF_i = < r_i - r_i^{av} >$$

where r_i stands for the i-th position of C_α atoms and r_i^{av} stands for the averaged i-th position of C_α atoms. < > indicates the statistical average.

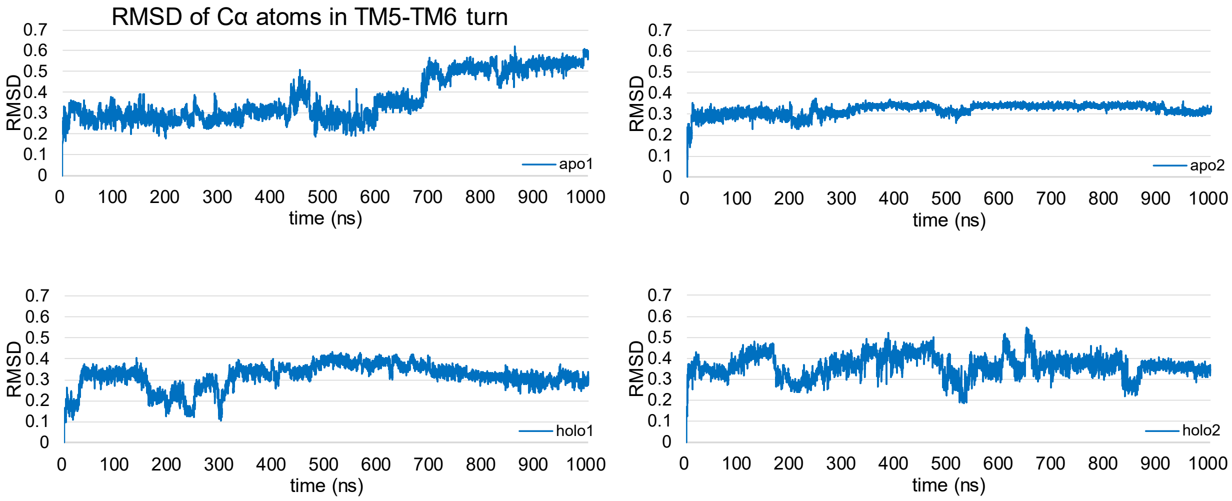

B) RMSD in TM5–TM6 turn

The RMSDs in the turn region between TM5 and TM6 in apo1, apo2, holo1, and holo2.

Figure S2 Conformational changes of CXCR4 structure by eliminating the ligand

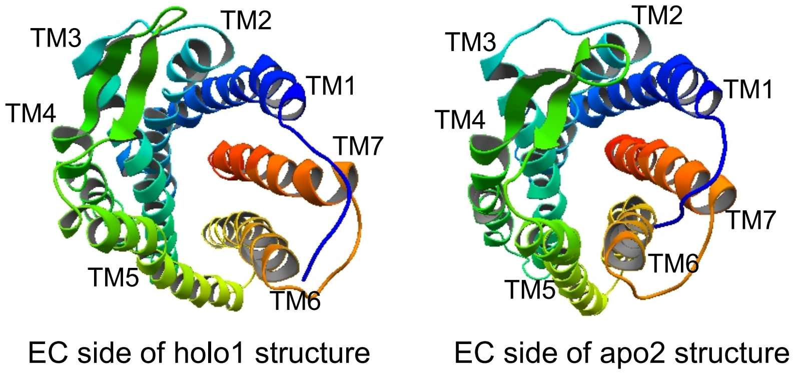

A) Structural comparison of apo form with holo form in the EC side

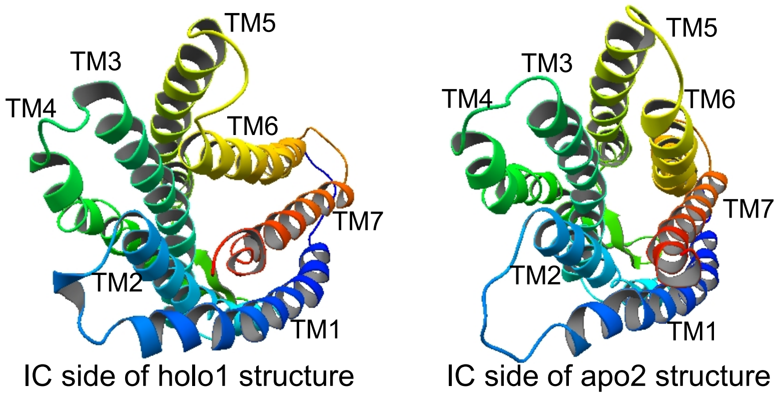

B) Structural comparison of apo form with holo form in the IC side

A, B) The snapshots at 1000 ns in the CXCR4 holo1 and apo2 structures are shown, where the CXCR4 is gradually colored from blue (N-terminus) to red (C-terminus). The left and right figures show the snapshots in holo and apo forms, respectively.

Figure S3 Clustering results of DIO vectors

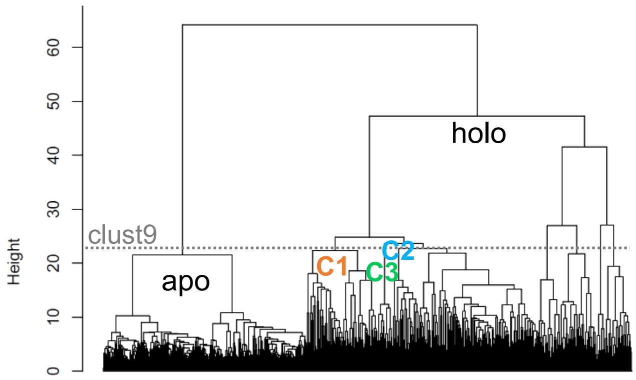

A) apo1–holo1

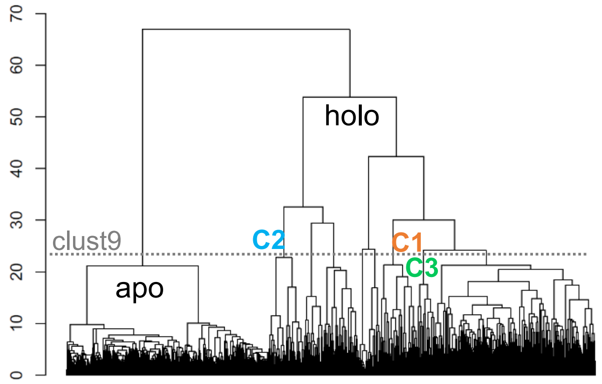

B) apo1–holo2

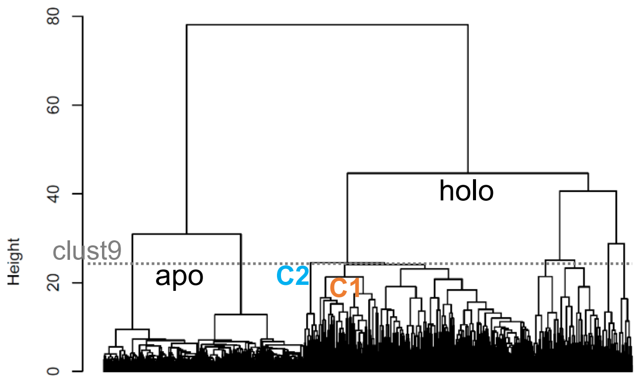

C) apo2–holo1

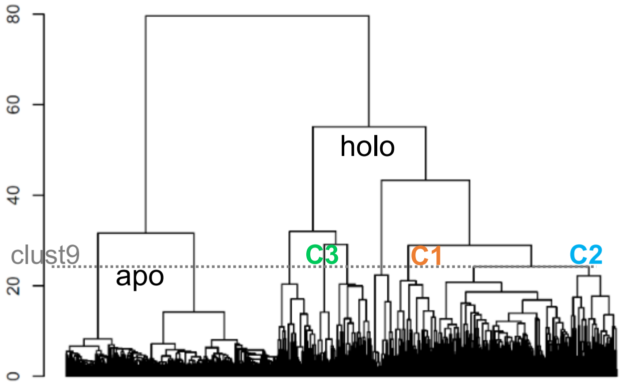

D) apo2–holo2

A, B) The dendrograms in the clustering of DIO vectors in apo1–holo1 and apo1–holo2 data, respectively. The DIO vectors were obtained in the inspections by apo1-trained AE for apo1, holo1, and holo2 data.

C, D) The dendrograms in the clustering of DIO vectors in apo2–holo1 and apo2–holo2 data, respectively, which were based on the inspections by apo2-trained AE for apo1, holo1, and holo2 data.

In all figures, C1, C2, and C3 indicate the cluster IDs shown in Table S2. Clustering was performed using R program [1].

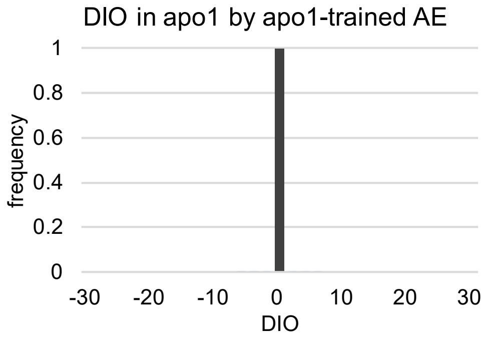

E)

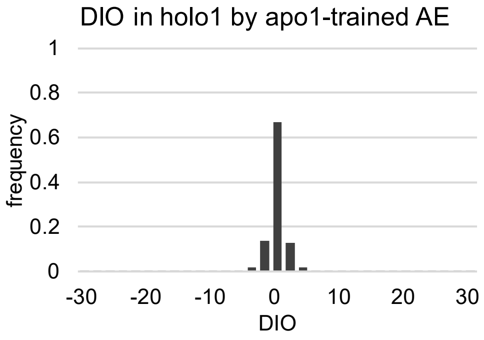

F)

E, F) Histograms of DIOs in apo1 (E) and holo1 (F) by apo1-trained AE.

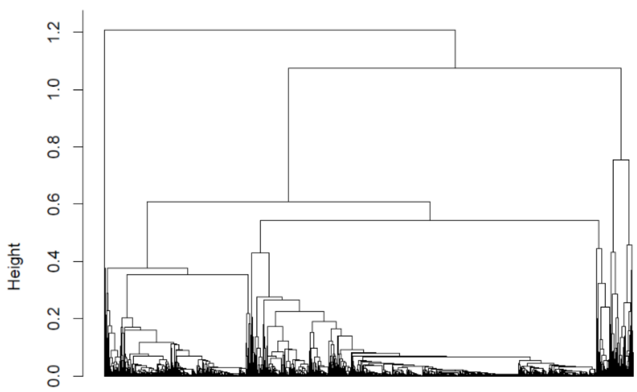

G) input vectors

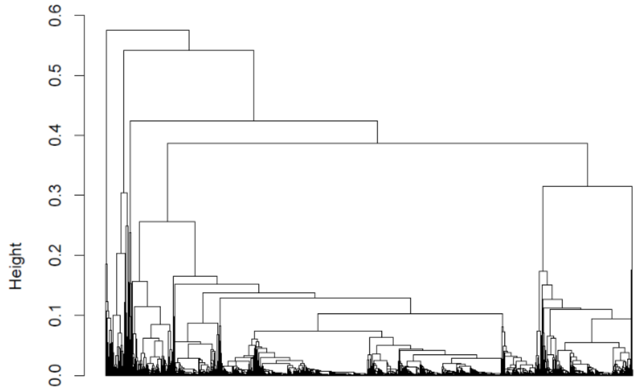

H) output vectors

G, H) The dendrograms in the clustering of the input vectors in apo1–holo1 data, and that of the output vectors by apo1-trained AE in apo1–holo1 data, respectively.

Figure S4 Sequence alignment of the CXCR family

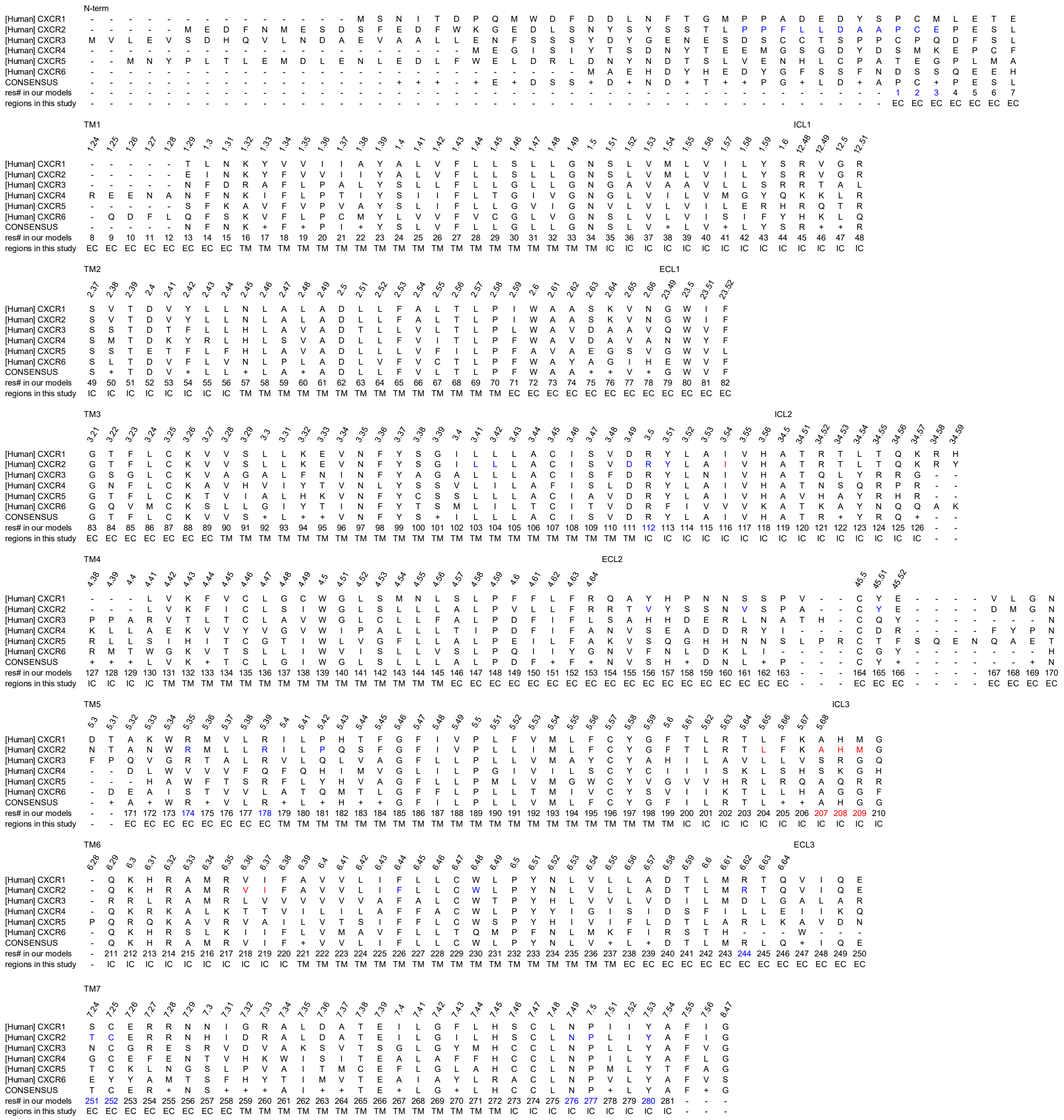

The sequence alignment of the CXCR protein family was constructed using the GPCRdb web server (https://gpcrdb.org/ [2]). Two types of lines were added: the lines with “res# in our models,” which indicate the residue number in our models of the CXCR4 structures, and the lines with “regions in this study,” which indicate the EC, TM, and IC regions in the CXCR4 structure defined in this study (see Figure 2). The residues shown in blue and red in CXCR2 lines are essential residues in ligand binding and conformational changes, and the signaling to G protein, respectively, as described in the literature on the EM structures of CXCR2 [3]. If the corresponding residues in CXCR4 were defined as Lead and/or Accm residues, the numbers in “res# in our models” lines were shown in the same color as those in CXCR2.

Figure S5 Relationships between the averages of residue–residue distances and the variances of the distances

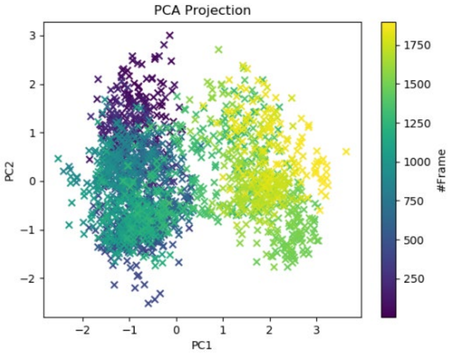

A) apo1

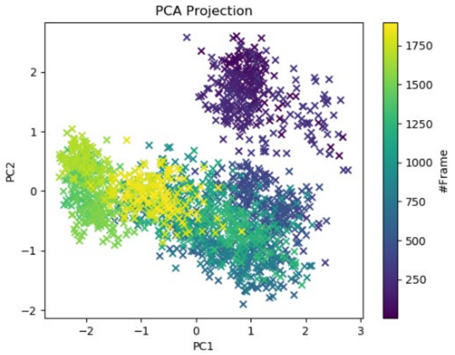

B) apo2

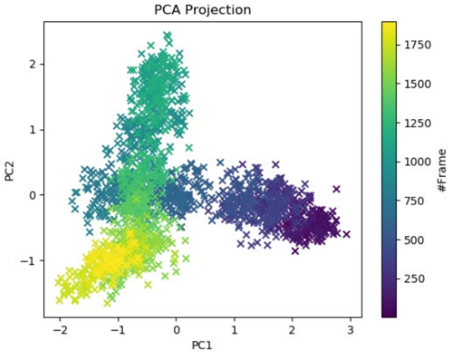

C) holo1

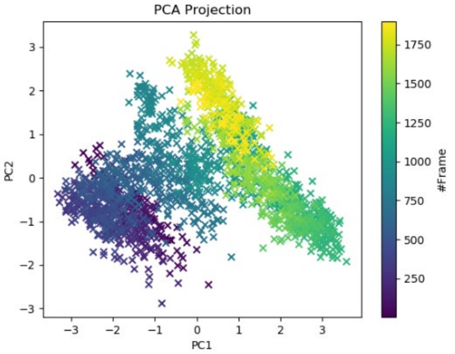

D) holo2

A–D) Principal component analyses (PCAs) of the MD trajectories in apo1, apo2, holo1, and holo2. PCA was performed using the MODE-TASK program [4].

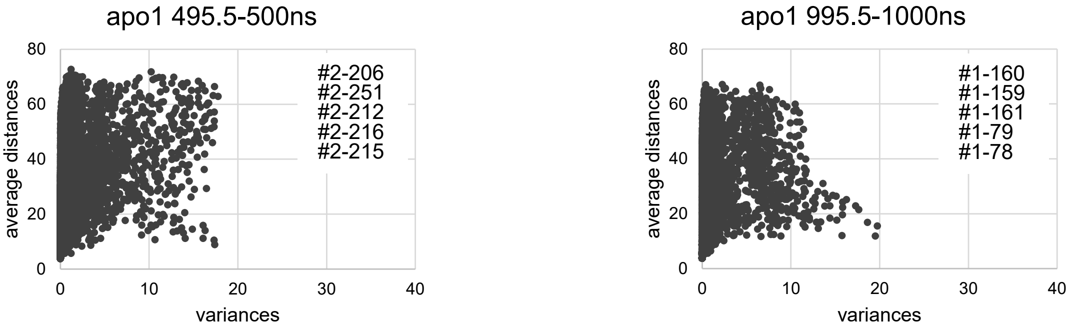

E) apo1

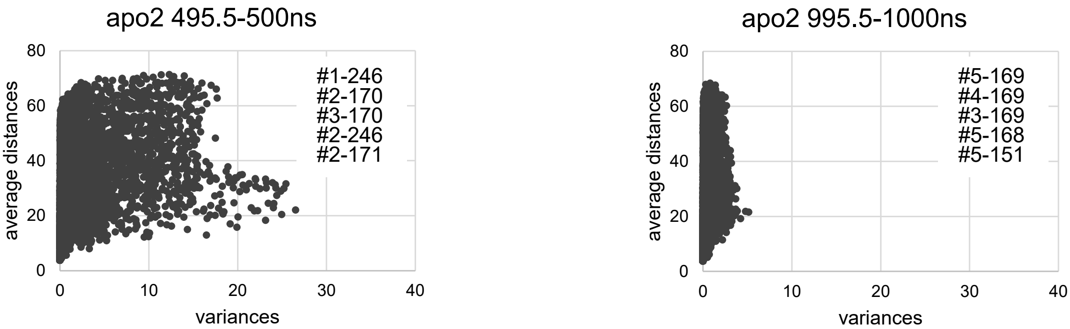

F) apo2

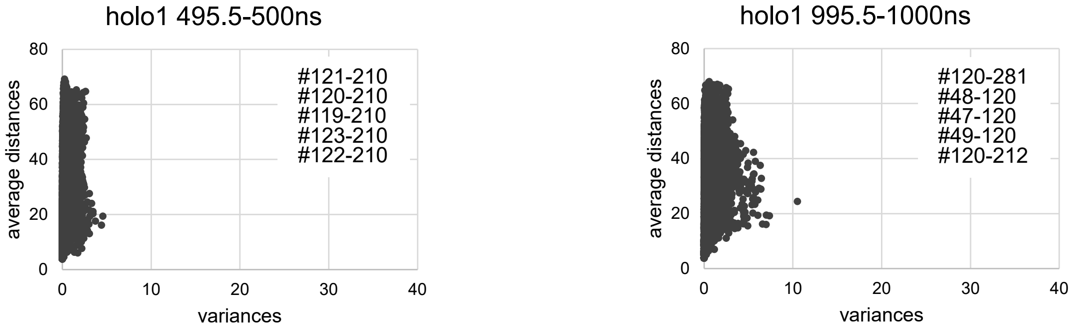

G) holo1

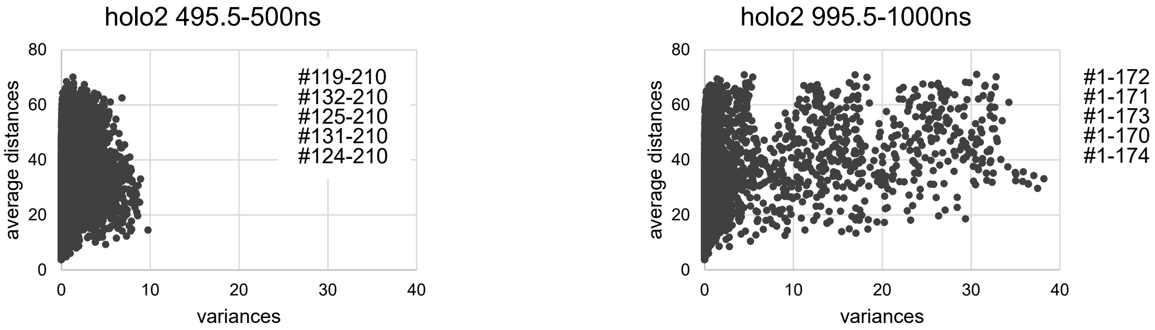

H) holo2

E–H) The correlations between the averages of residue–residue distances and the variances of the distances in two ranges of time steps, from 495.5 ns to 500 ns (left panels) and from 995.5 ns to 1,000

ns (right panels), are shown. The residue pairs with the five largest variances are shown in the upper-right regions.
E) In apo1 data, the residue pairs between the N-terminus and the turn between TM2 and TM3, TM4 and TM5, or TM6 and TM7 in the extracellular (EC) region and between the N-terminus and the intracellular (IC) region in TM5 and TM6 had large variances, i.e., largely fluctuated with correlation.
F) In apo2, particularly in the time steps from 495.5 ns to 500 ns, the residue pairs between N-terminus and the turn between TM4 and TM5 or TM6 and TM7 in EC region had large variances.
G) In holo1, particularly in the time steps from 995.5 ns to 1,000 ns, the pairs of residues located in IC regions, such as the turns between TM1 and TM2, TM3 and TM4, and TM5 and TM6, and C-terminus, had large variances.
H) In holo2, particularly in the time steps from 995.5 ns to 1,000 ns, the residue pairs between the N-terminus and the residues in the EC regions in TM4 and TM5, had large variances.

Most of these residue pairs were located around the TM turns. In addition, many of them were located in the EC regions in the apo (apo1 and apo2) form, while they were located in IC regions in the holo (holo1 and holo2) form. These findings suggest that ligand binding led to a decrease in correlative motions in the EC regions in the holo form, whereas ligand elimination led to an increase in the correlative motions in the EC regions in the apo form because of the large fluctuations of the turn between TM4 and TM5. The following are the exceptions: the motions in EC (the N-terminus) and IC regions (around the TM5–TM6 turn) for 495.5–500 ns in apo1 data and those in EC regions (the N-terminus and around the TM4–TM5 turn) for 995.5–1,000 ns in the holo2 data. We believe that the former may be related to the structural transition as described in the Results section and the latter may show large fluctuations of the receptor residues that were involved in the fluctuations of the ligand. Thus, the PCA-like analyses mainly represent the large and correlative fluctuations within the EC or IC regions, which suggests that these results correlate with those of RMSFs.

Figure S6 The comparison of DCCMs between the apo and holo forms

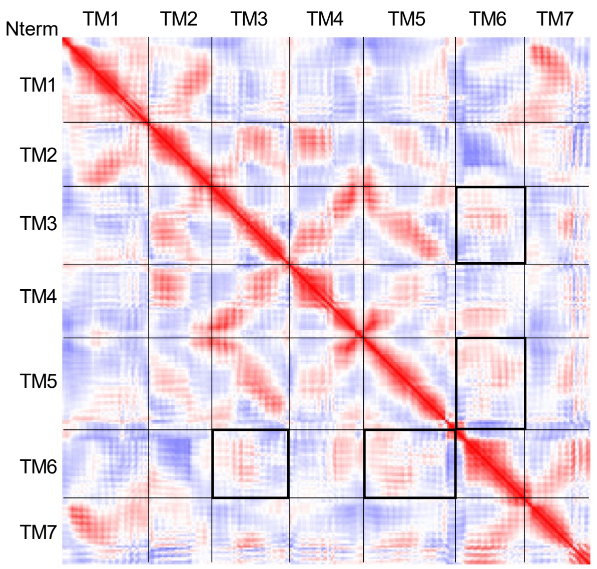

A) apo1

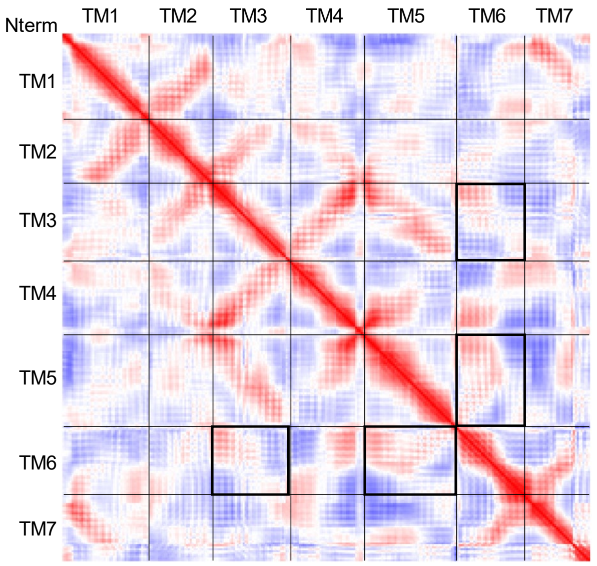

B) apo2

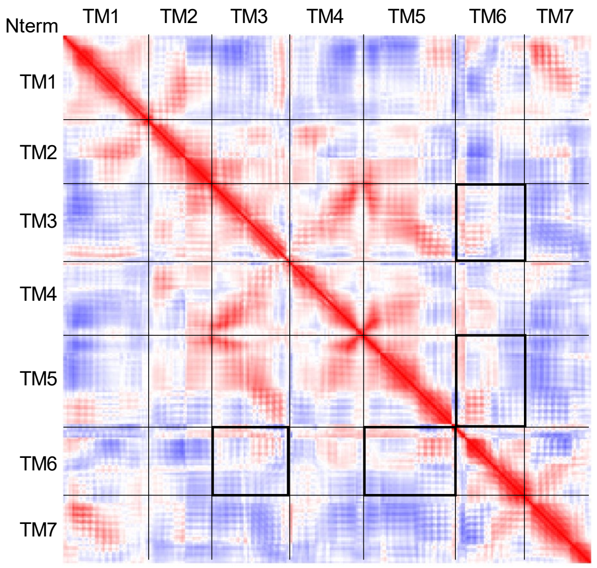

C) holo1

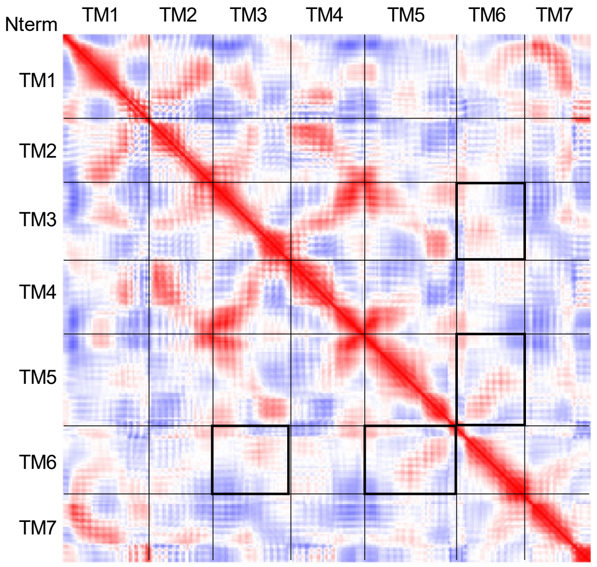

D) holo2

The DCCMs based on the motion of Cα atoms in receptor proteins were calculated by "covar" function in GROMACS [5]. The residue pairs with different signs of the correlations between apo (apo1 and apo2) and holo (holo1 and holo2) forms were examined for every pair of TM regions. The two pairs of TM regions, TM3 and TM6 and TM5 and TM6, which are marked with squares, included two largest numbers (259 and 211, respectively) of residue pairs with different signs of the correlations. In the two regions, particularly the N-terminus of these TM helices, the number of residue pairs with positive correlations (red dots) are larger in the apo form than in the holo form,

indicating that the correlative fluctuations were more observed in the apo form than in the holo form. These N-terminus regions were involved in ligand binding (in TM3) and conformational changes (in TM5 and TM6) in the holo form. Thus, the DCCMs suggest that ligand elimination and conformational reformation by elimination leads to correlative fluctuations between TM3 and TM6 and between TM5 and TM6, particularly in their N-terminus regions.

The other pairs of TM regions did not show clear differences, e.g., the pair of TM1 and TM7 regions, including the third largest 144 residue pairs with different signs; that of TM2 and TM4, including the fourth largest 140 residue pairs with different signs; and that of TM2 and TM3, including the fifth largest 135 residue pairs with different signs. Therefore, we focused only on the largest two regions in this study. Of note, the definition of TM regions is the same as that described in the Results section.

Using the values of DCCM, a network can be generated with the correlated residues as community nodes and the relationships between communities as edges. The generated network can then be ranked by betweenness centrality to order the essential residues. We compared these results with the experimental results and calculated the precision and recall by using the betweenness with the maximum precision as the threshold. As a result, the recall and precision were 0.32 and 0.10, respectively, which confirms the superiority of our AE-based method (0.56 and 0.14, respectively) in this comparison. The results of this comparison with the AE-based method are shown in the "Validation of AE-based results by comparing with experimental results" subsection. The Bio3D-2.3 package in R was used for this analysis [6].

Table S1 Intra-receptor protein contacts

The regions forming contacts [1]		The number of contacts [2]				The ratio of smaller to larger number of contacts [3]				
		apo1	apo2	holo1	holo2	small/large	s1/l1	s2/l1	s1/l2	s2/l2
TM1_EC	TM7_EC	2501	2455	2258	2309	holo/apo	0.90	0.92	0.92	0.94
TM6_TM	TM7_TM	2201	2621	46	39	holo/apo	0.02	0.02	0.02	0.01
TM3_EC	TM5_EC	1900	1900	1900	1900	apo/holo	1.00	1.00	1.00	1.00
TM2_IC	TM2_IC	1788	375	481	887	holo/apo	0.27	0.50	1.28	2.37
TM2_EC	TM5_EC	1574	1536	1517	1198	holo/apo	0.96	0.76	0.99	0.78
TM3_TM	TM4_IC	1550	1359	1748	1633	apo/holo	0.89	0.78	0.95	0.83
TM3_TM	TM4_EC	957	877	668	911	holo/apo	0.70	0.95	0.76	1.04
TM1_EC	TM7_TM	712	477	522	481	holo/apo	0.73	0.68	1.09	1.01
TM3_TM	TM4_EC	537	742	1123	653	apo/holo	0.48	0.66	0.82	1.14
TM3_TM	TM5_TM	523	1017	458	243	holo/apo	0.88	0.46	0.45	0.24
TM1_IC	TM2_TM	498	503	707	568	apo/holo	0.70	0.71	0.88	0.89
TM2_TM	TM3_TM	442	323	662	339	apo/holo	0.67	0.49	1.30	0.95
TM2_IC	TM4_IC	359	403	296	263	holo/apo	0.82	0.73	0.73	0.65
TM3_IC	TM4_IC	265	245	229	5	holo/apo	0.86	0.02	0.93	0.02
TM1_EC	TM1_EC	245	236	66	127	holo/apo	0.27	0.52	0.28	0.54
TM4_EC	TM5_EC	231	196	114	82	holo/apo	0.49	0.35	0.58	0.42
TM5_EC	TM6_EC	230	338	0	0	holo/apo	0.00	0.00	0.00	0.00
TM7_EC	TM7_EC	223	135	206	228	apo/holo	1.08	0.66	0.98	0.59
TM5_EC	TM5_EC	204	155	195	34	holo/apo	0.96	0.17	1.26	0.22
TM1_TM	TM7_IC	192	552	582	635	apo/holo	0.33	0.95	0.30	0.87
TM7_TM	TM7_IC	144	150	654	1091	apo/holo	0.22	0.23	0.13	0.14
TM1_TM	TM2_TM	133	169	190	257	apo/holo	0.70	0.89	0.52	0.66
TM3_IC	TM6_IC	14	29	309	341	apo/holo	0.05	0.09	0.04	0.09
TM2_TM	TM7_IC	2	23	407	281	apo/holo	0.00	0.06	0.01	0.08
TM1_EC	TM7_EC	2501	2455	2258	2309	holo/apo	0.90	0.92	0.92	0.94
TM6_TM	TM7_TM	2201	2621	46	39	holo/apo	0.02	0.02	0.02	0.01

1) The intra-receptor protein contacts were formed between these two regions. For example, the pair of TM1_EC and TM7_EC indicates that the contacts were formed between the residues in the extracellular (EC) region on the helix 1 and the EC region on the helix 7.

2) The number of contacts observed in apo1, apo2, holo1, and holo2 structures are shown.

3) The ratio of smaller contacts to larger contacts. If the sum of the numbers of contacts in apo1 and apo2 are larger than that in holo1 and holo2, then the “small/large” shows “holo/apo”, and the ratios of holo1 to apo1, holo2 and apo1, holo1 and apo2, and holo2 and apo2, are shown in the columns “s1/l1”, “s2/l1”, “s1/l2”, and “s2/l2”, respectively.

Table S2 Lead and Accm residues in CXCR4

Apo1-holo1	rec/lig	Lead (25 residues)	Accm (37 residues)
Cluster 1	rec-rec	1-5(TM1_EC), 157(TM4_EC), 248(TM6_EC), 249-250(TM7_EC)	6-9(TM1_EC), 12(TM1_EC), 158-160(TM4_EC), 237(TM6_TM), 240-247(TM6_EC), 251(TM7_EC), 253-255(TM7_EC)
	rec-lig	1-3(TM1_EC), 249(TM7_EC)	-
	Functional roll: the interaction with, or the signaling from, the ligand.		
Cluster 2	rec-rec	118(TM3_IC), 208-210(TM5_IC), 211-214(TM6_IC)	112(TM3_IC), 205-207(TM5_IC), 215(TM6_IC), 217(TM6_IC), 229(TM6_TM)
	rec-lig	-	206(TM5_IC) 208-210(TM5_IC), 211-214(TM6_IC)
	Functional roll: the signaling to the G protein.		
Cluster 3	rec-rec	273(TM7_IC), 275-281(TM7_IC)	272(TM7_TM), 274(TM7_IC)
	rec-lig	-	-
	Functional roll: the transmission of the signal to the cell.		

Apo1-holo2	rec/lig	Lead (43)	Accm (43)
Cluster 1	rec-rec	1-4(TM1_EC), 249-250(TM7_EC)	5-9(TM1_EC), 240-248(TM6_EC)
	rec-lig	-	-
	Functional roll: the interaction with, or the signaling from, the ligand.		
Cluster 2	rec-rec	-	-
	rec-lig	1-2(TM1_EC), 4-8(TM1_EC), 10-11(TM1_EC), 238-248(TM6_EC), 249-258(TM7_EC), 259-260(TM7_TM)	9(TM1_EC), 12-15(TM1_EC), 171-172(TM5_EC), 174-178(TM5_EC), 179-180(TM5_TM), 205(TM5_IC), 217(TM6_IC), 231-237(TM6_TM), 261-265(TM7_TM)
	Functional roll: the interaction with, or the signaling from, the ligand to the TM region, and the conformational changes of TM5, TM6, and TM7.		
Cluster 3	rec-rec	206-210(TM5_IC), 211-215(TM6_IC)	-
	rec-lig	206-210(TM5_IC), 211-212(TM6_IC)	214-215(TM6_IC)
	Functional roll: the signaling to G the protein.		

Apo2-holo1	rec/lig	Lead (25)	Accm (23)
Cluster 1	rec-rec	1-5(TM1_EC), 122(TM4_IC), 159(TM4_EC), 178(TM5_EC), 206-210(TM5_IC), 211-214(TM6_IC)	6-8(TM1_EC), 112(TM3_IC), 118(TM3_IC), 179(TM5_TM), 205(TM5_IC), 215(TM6_IC), 217(TM6_IC), 222(TM6_TM)
	rec-lig	1-3(TM1_EC)	4-5(TM1_EC), 7(TM1_EC), 118(TM3_IC), 122(TM4_IC), 179(TM5_TM), 206(TM5_IC), 208-210(TM5_IC), 211-214(TM6_IC)
	Functional roll: the interaction with, or the signaling from, the ligand, and the signaling to the G protein.		
Cluster 2	rec-rec	272(TM7_TM), 273(TM7_IC), 276-281(TM7_IC)	274-275(TM7_IC)
	rec-lig	-	-
	Functional roll: the transmission of the signal to the cell.		

Apo2-holo2	rec/lig	Lead (56)	Accm (41)
Cluster 1	rec-rec	1-4(TM1_EC), 249(TM7_EC)	5-6(TM1_EC), 8-9(TM1_EC), 240-242(TM6_EC), 244-248(TM6_EC), 253-255(TM7_EC), 261(TM7_TM)
	rec-lig	1-2(TM1_EC)	-
	Functional roll: the interaction with, or the signaling from, the ligand.		
Cluster 2	rec-rec	39(TM1_IC), 43-45(TM1_IC), 46-47(TM2_IC), 206-210(TM5_IC), 211-215(TM6_IC), 275-281(TM7_IC)	36-38(TM1_IC), 40-42(TM1_IC), 273(TM7_IC)
	rec-lig	46(TM2_IC), 206-210(TM5_IC), 211-212(TM6_IC)	45(TM1_IC), 47(TM2_IC), 213-215(TM6_IC)
	Functional roll: the signaling to G the protein, and the transmission of the signal to the cell.		

Cluster 3	rec-rec	-	-
	rec-lig	3-8(TM1_EC), 10-11(TM1_EC), 239-248(TM6_EC), 250-258(TM7_EC), 259-261(TM7_TM)	12(TM1_EC), 14-15(TM1_EC), 231-233(TM6_TM), 235-237(TM6_TM), 238(TM6_EC), 249(TM7_EC), 262(TM7_TM), 265(TM7_TM)
	Functional roll: the interaction with, or the signaling from, the ligand to the TM region, and the conformational changes of TM6 and TM7.		

1) The residue numbers (e.g. 1-5), the transmembrane helix number (from TM1 to TM7), and the region on the helix (extracellular (EC), transmembrane (TM), or intercellular (IC) region) are shown.
2) The residue numbers in “rec–rec” and “rec–lig” rows indicate the receptor residue numbers in receptor–receptor and receptor–ligand residue pairs, respectively.
3) The information only in the holo form without the ninth cluster is shown.
4) The total number of Lead and Accm residues in each combination without the overlaps is 55, 71, 37, or 76 for apo1–holo1, apo1–holo2, apo2–holo1, or apo2–holo2 combination, respectively.

Table S3 The essential residues involved in the ligand and G protein binding in CXCR2

CXCR2 [1)]	CXCR4 [2)]	Interactions [3)]	Signal [4)]	apo1-holo1 [5)]	apo1-holo2 [5)]	apo2-holo1 [5)]	apo2-holo2 [5)]
P38	1(TM1_EC)	Ligand	Ligand	Lead	Lead	Lead	Lead
C39	2(TM1_EC)	Ligand	Ligand	Lead	Lead	Lead	Lead
E40	3(TM1_EC)	Ligand	Ligand	Lead	Lead	Lead	Lead
L134(3.40)	103(TM3_TM)	TM5conf	Ligand				
D143(3.49)	111(TM3_TM)	TM5conf	Ligand				
R144(3.50)	112(TM3_IC)	TM5conf	Ligand	Accm		Accm	
Y145(3.51)	113(TM3_IC)	TM5conf	Ligand				
Y197(ECL2)	165(TM5_EC)	Ligand	Ligand				
R208(5.35)	174(TM5_EC)	Ligand	Ligand		Accm		
R212(5.39)	178(TM5_EC)	Ligand	Ligand		Accm	Lead	
P223(5.50)	181(TM5_TM)	TM5conf	Ligand				
F260(6.44),	226(TM6_TM)	TM5conf	Ligand				
W264(6.48)	230(TM6_TM)	TM5conf	Ligand				
R278(6.62)	244(TM6_EC)	Ligand	Ligand	Accm	Lead/Accm		Lead/Accm
T285(7.24)	251(TM7_EC)	Ligand	Ligand	Accm	Lead		Lead
I148(3.53)	116(TM3_IC)	G protein	G protein				
L238(5.65)	204(TM5_IC)	G protein	G protein				
A241(ICL3)	207(TM5_IC)	G protein	G protein	Accm	Lead	Lead	Lead
H242(ICL3)	208(TM5_IC)	G protein	G protein	Lead/Accm	Lead	Lead/Accm	Lead
M243(ICL3)	209(TM5_IC)	G protein	G protein	Lead/Accm	Lead	Lead/Accm	Lead
V252(6.36)	218(TM6_IC)	G protein	G protein				
I253(6.37)	219(TM6_IC)	G protein	G protein				
N310(7.49)	276(TM7_IC)	TM7conf	G protein	Lead		Lead	Lead
P311(7.50)	277(TM7_IC)	TM7conf	G protein	Lead		Lead	Lead
Y314(7.53)	280(TM7_IC)	TM7conf	G protein	Lead		Lead	Lead

1) The residue numbers in the CXCR2 structure, with the generic residue numbers based on the GPCRab numbering scheme [7] in parentheses, are shown.
2) The residue number in our CXCR4 model, which corresponds with the residue in the CXCR2 line based on the sequence alignment, is shown.
3) The interactions which the residues were involved in, are shown. The residues with "Ligand" interacted with ligand or were involved in the signaling from the ligand, those with "TM5conf" were involved in the conformational changes of TM5 and TM6 or the synergetic rearrangements of TM3, those with "TM7conf" were involved in the conformational changes of TM7 for arrestin recognition, which might lead the transmission of the signal to the cell, and those with "G protein" were involved in the signaling to the G protein.
4) "Ligand" and "G protein" indicate that the interactions were involved in the signaling from ligand and that to G protein, respectively.
5) The "Lead" and "Accm" in apo1–holo1, apo1–holo2, apo2–holo1, and apo2–holo2 columns indicate that the residues in the CXCR4 model were detected as Lead or Accm residues.

References

1. R Core Team. R: A language and environment for statistical computing. R Foundation for Statistical Computing, Vienna, Austria. (2020).
2. Pándy-Szekeres, G. *et al.* GPCRdb in 2018: Adding GPCR structure models and ligands. *Nucleic Acids Res.* **46**, D440–D446 (2018).
3. Liu, K. *et al.* Structural basis of CXC chemokine receptor 2 activation and signalling. *Nature* **585**, 135–140 (2020).
4. Ross, C. *et al.* MODE-TASK: Large-scale protein motion tools. *Bioinformatics* **34**, 3759–3763 (2018).
5. Abraham, M. J. *et al.* Gromacs: High performance molecular simulations through multi-level parallelism from laptops to supercomputers. *SoftwareX* **1–2**, 19–25 (2015).
6. Grant, B. J., Skjærven, L. & Yao, X. Q. The Bio3D packages for structural bioinformatics. *Protein Sci.* **30**, (2021).
7. Isberg, V. *et al.* Generic GPCR residue numbers - Aligning topology maps while minding the gaps. *Trends in Pharmacological Sciences* vol. 36 22–31 (2015).
